# Increasing sun protection behaviors among Iranian farmworkers: a call for action

**DOI:** 10.15171/hpp.2017.02

**Published:** 2016-12-18

**Authors:** Brian Martin, Vinayak K. Nahar, Amanda K. Hutcheson, Javier F. Boyas, Manoj Sharma

**Affiliations:** ^1^Biomedical Professionals, Lincoln Memorial University, Harrogate, TN, USA; ^2^Department of Dermatology, School of Medicine, University of Mississippi Medical Center, Jackson, MS, USA; ^3^Department of Health, Physical Education, and Exercise Science, School of Allied Health Sciences, Lincoln Memorial University, Harrogate, TN, USA; ^4^Department of Health Science, College of Human Environmental Sciences, The University of Alabama, Tuscaloosa, AL, USA; ^5^Department of Social Work, School of Applied Sciences, University of Mississippi, University, MS, USA; ^6^Behavioral & Environmental Health, School of Public Health, Jackson State University, MS, USA; ^7^College of Health Sciences, Walden University, Minneapolis, MN, USA

## Dear Editor,

According to the World Health Organization (WHO), every year between two and three million non-melanoma skin cancers and 132 000 malignant melanoma cases occur worldwide.^1^ Incidence rates have been increasing in several countries, including Iran, where skin cancers are the most common type of cancer.^2^ A recent 10-year retrospective study conducted in Iran found a higher incidence of melanoma in male farmworkers.^3^ That is because among occupations, outdoor workers and farm workers consistently have the highest rates of skin cancers, due largely to high exposure to ultraviolet radiation (UVR).^4,5^ Some estimates suggest that sun exposure is the cause of 90% of all melanomas.^6^ High levels of sun exposure early in life have also been implicated in increasing the chances of developing skin cancers, and farm workers commonly begin heavy sun exposure at a young age.^4^

Agriculture is a high-risk occupation, and because of this, farm workers are exposed to many short-term dangers, which may distract them from the long-term dangers of skin cancer.^6^ Avoiding direct sunlight is a key strategy to skin cancer prevention, but this is a daunting task for farmworkers who must work in a field with little, to no sun protection.^6^ Because of this, sunscreen application of an appropriate sun protection factor (SPF) level is imperative. Studies show that regular sunscreen usage reduced the rate of skin cancers in the population by up to 9.3% and melanoma up to 14%.^7^ Unfortunately, farm workers consistently have negative attitudes toward sunscreen usage. Expressed concerns include its messy nature, inconvenience in application, need for reapplication, association with being feminine, and fear of being ridiculed.^6,8^ Therefore, in future interventions targeting farm workers, it is necessary to emphasize the effectiveness of protective measures and concurrently address the elimination of barriers, which may keep farm workers from engaging in sun protection practices. To encourage sunscreen use, a practical strategy may be to have sunscreen dispensers set up throughout the work sites.^9^ This may encourage farm workers to apply it and serve as a reminder that they should apply sunscreen multiple times to better protect themselves against the harms of UVR. It is also more likely that farm workers will apply sunscreen if this behavior is supported by fellow farm workers and supervisors.^10^

Sun protection products are not commonly advertised in Iran,^11^ compounding the issue of nonuse. Most individuals are directed to use sunscreen by either a doctor or a pharmacist.^11^ Furthermore, if patients have a question regarding a skin protection product, they are more likely to see a pharmacist due to either expense or because they do not think it is worth scheduling an appointment with a doctor to discuss this issue.^12^ This is especially problematic when, as Movaffagh et al found, pharmacists scored less than 50% when tested on their knowledge about skin cancer and sunscreen usage.^12^ This suggests that there is an urgent need of skin cancer educational interventions targeting healthcare providers in Iran, especially those who interface with farm workers.

Another important skin protection strategy is protective clothing such as hats, sunglasses or long sleeves. Male farm workers are more likely to wear a hat than they are to apply sunscreen.^6^ The most common areas to develop skin cancer among farm workers are the nose and cheek, followed by the ear, lips and scalp, emphasizing the importance of wearing a wide-brimmed hat.^4^ Many farm workers were found to wear hats as their primary means of skin protection, but did not wear a hat which provided adequate protection.^13^ Long sleeves were already used by farm workers in one study to protect themselves from bugs and dust,^8^ although this was not observed in all communities.^14^ Common reasons provided for not wearing protective clothing were that they were too hot, a distraction, or it simply was convenient not to wear them.^6^ However, a relatively high number stated that they wore protective clothing when using agricultural chemicals,^15^suggesting that protective measures are taken when risk is deemed high enough. Education programs which express the dangers of excessive sunlight exposure and the necessity of skin protection have been proven effective in changing behavior.^4^

In addition to skin protective strategies, previous research has identified a lack of perceived susceptibility and severity for skin cancer among Iranian farmworkers.^16,17^ Studies using behavioral theories such as Protection Motivation Theory (PMT) have identified unacceptable levels of threat appraisal for skin cancer among Iranian farm workers, including a study by Tazval et al,^16^ which identified that approximately 60% of farm workers surveyed had very low perceived threat levels. Another study conducted among Iranian farm workers in a rural community found that perceived susceptibility, rewards, and self-efficacy were the strongest and most positive, predictors of skincare protective behaviors.^17^ Findings from research utilizing theories suggest that health care providers and health promotion practitioners should focus on developing theoretically-based health education programs targeting skin cancer prevention for this population using health education theories such as PMT.^16,17^ Previous studies have successfully implemented PMT-based interventions to address sun protective behaviors among other Iranian populations; thus, suggesting the efficacy of using PMT or other value expectancy theories to develop a theory-based health education intervention for farmworkers.^18^

Another strategy that could be useful is training farm workers to serve as lay health promoters. In the past 25 years in the United States, the use of farm workers as para-professionals who provide health education and outreach services within their respective communities has been a widely accepted method of health promotion in agricultural industries with ethnic minority populations.^19^ In the United States, the model of “*Promotora de Salud*” (Health Promoters) has been widely used with Hispanic/Latino farm workers. This model has been successful because the people carrying out the duties of promoting safe health practices are fellow, trusted workers who can perhaps deliver health messages that are culturally appropriate,^20^ while simultaneously serving as an advocate, coach, supporter, and role model. Health messages may be more trustworthy when they are relayed by a fellow coworker. Although *lay health promoters* have been used mainly to address the risks from exposure to pesticides, it is possible that this model may be useful in promoting sun safe practices among farm workers in Iran. Employing fellow farm workers to promote sun safe practices may help other farm workers better recognize their susceptibility to skin cancer and the severity of skin cancer. Once farm workers understand the health threat posed by UVR and their vulnerability, they are more likely to take corrective action to better protect themselves.^16^

In summary, considering the high rates of skin cancers among Iranian farm workers, it is vital to increase their use of sun protection practices. Increasing sun protection strategies among this population should be a public health priority. Future interventions should focus on shifting farm workers’ attitudes towards skin cancer risk and prevention. Moreover, public health professionals should consider developing and testing theory-based educational interventions targeting the sun protection behaviors of farm workers. Much more attention has to also be given to developing practical ways of increasing sun protective strategies in the Iranian agricultural workplace. Doing so can help this vulnerable population decrease the occupational risks associated with elevated levels of UVR exposure, and by extension, reduce the high incidence rates of melanoma skin cancer among Iranian farm workers.

## Ethical approval

None to be declared.

## Competing interests

The authors declare that there is no conflict of interests.
